# Cytoarchitectonic mapping of the human dorsal extrastriate cortex

**DOI:** 10.1007/s00429-012-0390-9

**Published:** 2012-02-22

**Authors:** Milenko Kujovic, Karl Zilles, Aleksandar Malikovic, Axel Schleicher, Hartmut Mohlberg, Claudia Rottschy, Simon B. Eickhoff, Katrin Amunts

**Affiliations:** 1 C. & O. Vogt Institute for Brain Research, University of Düsseldorf, Düsseldorf, Germany; 2 Institute of Neuroscience and Medicine (INM 1, INM 2) and JARA, Translational Brain Medicine, Research Centre Jülich, 52425 Juelich, Germany; 3 Department of Psychiatry, Psychotherapy and Psychosomatics, RWTH Aachen University, Aachen, Germany; 4 Institute of Anatomy, Faculty of Medicine, University of Belgrade, Belgrade, Serbia

**Keywords:** Cytoarchitecture, Visual cortex, hOc3d, hOc4d, V3, V3a, Probabilistic maps, Human brain atlas

## Abstract

The dorsal visual stream consists of several functionally specialized areas, but most of their cytoarchitectonic correlates have not yet been identified in the human brain. The cortex adjacent to Brodmann area 18/V2 was therefore analyzed in serial sections of ten human post-mortem brains using morphometrical and multivariate statistical analyses for the definition of areal borders. Two previously unknown cytoarchitectonic areas (hOc3d, hOc4d) were detected. They occupy the medial and, to a smaller extent, lateral surface of the occipital lobe. The larger area, hOc3d, is located dorso-lateral to area V2 in the region of superior and transverse occipital, as well as parieto-occipital sulci. Area hOc4d was identified rostral to hOc3d; it differed from the latter by larger pyramidal cells in lower layer III, thinner layers V and VI, and a sharp cortex-white-matter borderline. The delineated areas were superimposed in the anatomical MNI space, and probabilistic maps were calculated. They show a relatively high intersubject variability in volume and position. Based on their location and neighborhood relationship, areas hOc3d and hOc4d are putative anatomical substrates of functionally defined areas V3d and V3a, a hypothesis that can now be tested by comparing probabilistic cytoarchitectonic maps and activation studies of the living human brain.

## Introduction

**Fig. 1:**
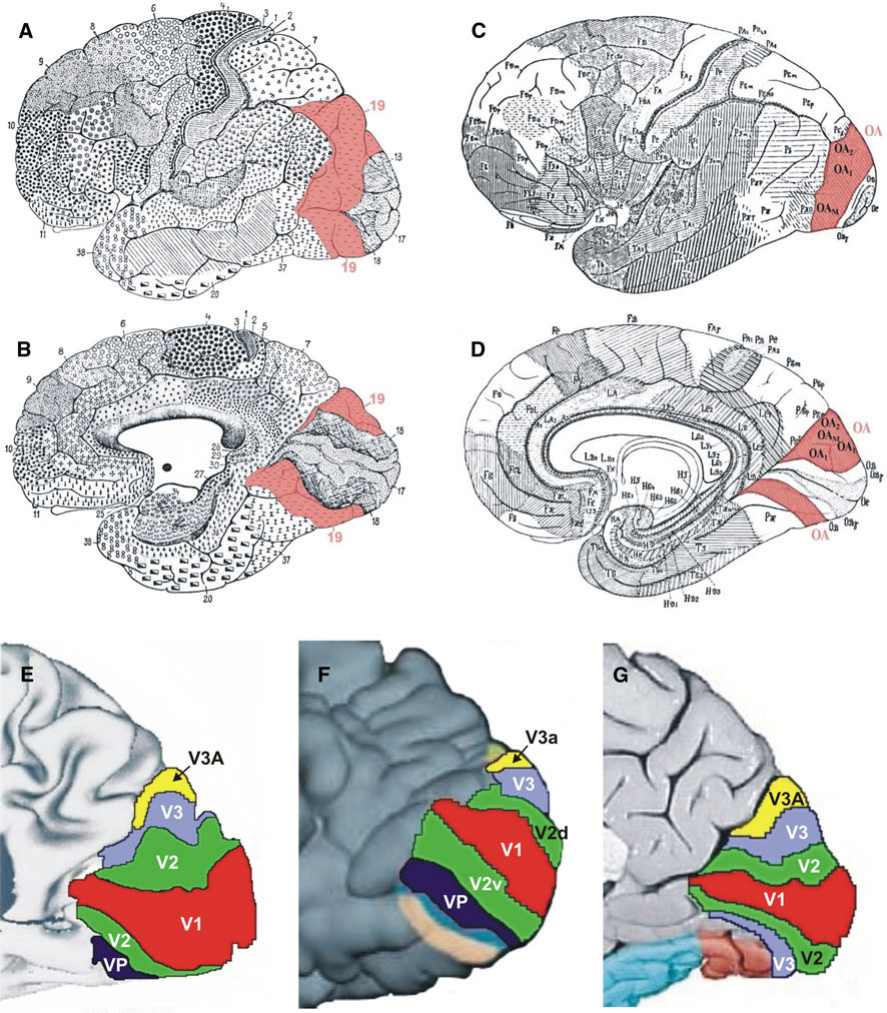
Cytoarchitectonic maps of Brodmann (1909) (*lateral view* **a** and *medial view* **b**) and von Economo and Koskinas (1925) (*lateral view* **c** and *medial view* **d**). Area 19, the putative region of interest, is labeled in *red*. Modified and adapted functional maps (*medial views* of the occipital lobe) of van Essen and Drury (1997) (**e**); Tootell and Hadjikhani (2001) (**f**) and Zeki 2003 (**g**) reveal the functional segregation of the visual cortex

Functional neuroimaging studies have revealed a complex segregation of the human visual cortex (DeYoe et al. 1996; Malach et al. 1995; Pitzalis et al. 2006; Reppas et al. 1997; Sereno et al. 1995; Tootell et al. 1995a, b; Tootell and Hadjikhani 2001; Watson et al. 1993; Wilms et al. 2005, 2010). A unique area V3, adjoining both the dorsal and the ventral parts of area V2 (BA 18/V2) has been proposed by Zeki (e.g., Zeki 1978, 2003). Other authors distinguished a dorsal (V3 (*d*)) and a ventral (VP or V3v) subarea of area V3 (van Essen and Drury 1997; Tootell and Hadjikhani 2001) (Fig. 1). The dorsal portion of area V3 in the macaque brain has been described as a functional unit of the dorsal stream, whereas the ventral V3 was seen as part of the ventral stream (Ungerleider and Mishkin 1982). According to the dorsal and ventral stream concept, areas of the dorsal stream would mainly be involved in the spatial awareness (e.g., during eye−hand coordination) and guidance of actions (e.g., reaching the objects). In contrast, areas of the ventral stream would be involved in the identification and recognition of objects (including their color and form) and faces (Ungerleider and Mishkin 1982).

The discussion also concerns the existence and the extent of area V3a along the lateral brain surface. Tootell et al. (1997) have performed a functional neuroimaging study and showed that area V3a was located almost in its full extent along the lateral surface of the occipital lobe, where it occupied even the most ventral parts. Two other studies noted a much more medial and dorsal location of area V3a (Fig. 1) along the posterior bank of the parieto-occipital sulcus (van Essen and Drury 1997; Zeki 2003). Area V3d has been activated by contrast and motion stimuli (Tootell et al. 1997). Area V3a was identified as motion sensitive (higher than area V3) (Tootell et al. 1997), 2D-shape sensitive (Grill-Spector et al. 1999) area involved in the extraction of 3D-structure from motion (Vanduffel et al. 2002).

Several anatomical studies employing cyto-, myelo- and receptorarchitctonic methods support the notion of a dorso-ventral segregation of the extrastriate cortex into dorsal and ventral areas beginning with the areas adjacent to BA 18/V2 (Clarke and Miklossy 1990; Zilles and Clarke 1997; Amunts et al. 2007; Rottschy et al. 2007; Eickhoff et al. 2008). Therefore, the classical tripartion of the visual cortex as indicated in Brodmann’s cytoarchitectonic map, which distinguishes a primary visual area 17 from two extrastriate areas 18 and 19 is not reflecting the organization of the visual cortex. However, even hundred years later there is no cytoarchitectonic map available that would reflect the spatial segregation of the complete visual cortex as suggested by recent functional imaging studies in the human brain of maps of the macaque cortex.

The aim of the present study was to further develop existing cytoarchitectonic maps of the striate and ventral extrastriate cortex (Amunts et al. 2000; Rottschy et al. 2007; Malikovic et al. 2007), and to map the extrastriate cortex dorsolateral to area BA18/V2 (Fig. 1). In order to avoid any unproven association with functionally defined areas and/or homologies to macaque brain, we applied a neutral nomenclature for the two new *cytoarchitectonic* areas, i.e., hOc3d and hOc4d (h = human, Oc = occipital cortex, d = dorsal).

## Materials and methods

### Post-mortem brains

Ten human post-mortem brains (5 male and 5 female, mean age: 66 years, age range: 37−85 years) were obtained through the body donor program of the Anatomical Institute of the University of Düsseldorf in accordance with the legal requirements (Table 1). The post-mortem delay was less than 24 h. One brain came from a subject with transitory motor deficits; all other subjects had no history of psychiatric or neurological diseases. Handedness of the subjects was unknown. The analyzed sample was the same as in our earlier anatomical studies of the visual cortex (Amunts et al. 2000; Malikovic et al. 2007; Rottschy et al. 2007).

**Table 1:** Sample of the present study. * F* – female;* M* – male

Case	Gender	Age (years)	Cause of death	Postmortem delay (h)	Fixation
1	F	79	Carcinoma of the bladder	24	Formalin
2	M	55	Rectal carcinoma	24	Formalin
3	M	68	Cardiovascular disease	16	Formalin
4	M	75	Acute glomerulonephritis	24	Bodian
5	F	59	Cardiorespiratory insufficiency	24	Formalin
6	M	54	Myocardial infarction	8	Formalin
7	M	37	Cardiac arrest	24	Formalin
8	F	72	Renal arrest	12	Bodian
9	F	79	Cardiorespiratory insufficiency	16	Bodian
10	F	85	Mesenteric infarction	14	Formalin

**Fig. 2:**
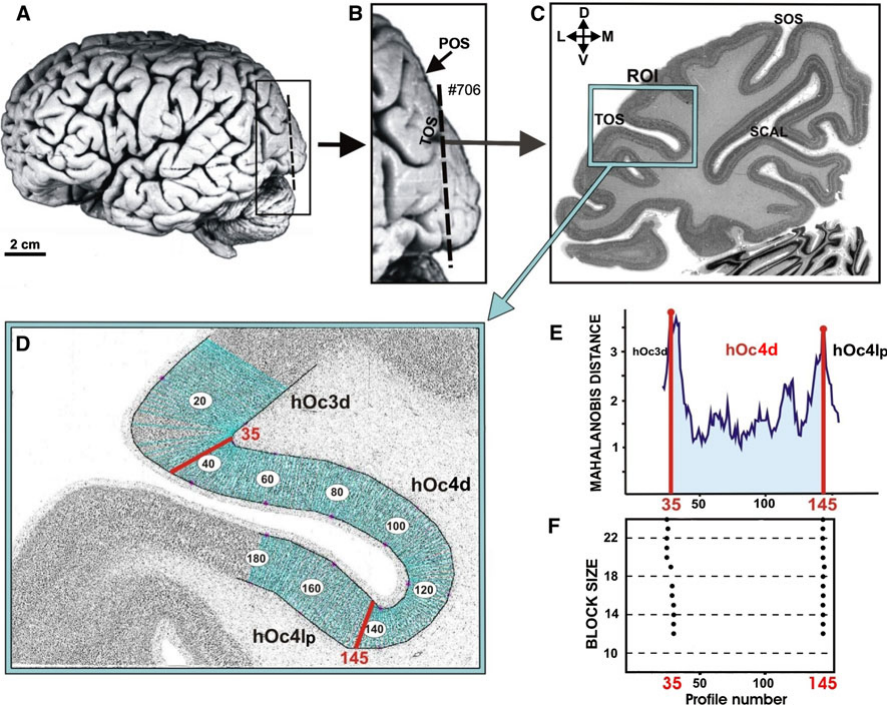
**a** Lateral view of post-mortem brain number 7, left hemisphere. The *dashed line* indicates the position of the coronal section shown in **c** and **d**. Major sulci on the dorsal surface of the occipital lobe (**b** and **c**). *TOS* transverse occipital sulcus, *POS* parieto-occipital sulcus, *SOS* superior occipital sulcus. **c** Cell body stained histological section. The *box marks* the ROI (region of interest) for the quantitative cytoarchitectonic analysis. Orientation: *D* dorsal, *V* ventral, *L* lateral, *M* medial. The positions of the GLI profiles within the ROI are marked in the inverted GLI image (**d**). Extent of areas hOC3d and hOc4d as defined by the cytoarchitectonic analysis (**f**). The Mahalanobis-distance function for the blocksize *b* = 24 (**e**). Scatterplot of the location of significant maxima (*black dots*) over all examined blocksizes (**f**). The location of borders does not vary in dependence on block size

The brains were fixed in 4% formalin or Bodian’s fixative for several months: they were suspended on the basilar/vertebral arteries to minimize distortions in brain shape during the fixation process. MR imaging of each post-mortem brain was performed after fixation with a Siemens 1.5-T scanner (Erlangen, Germany) using a T1-weighted 3D FLASH sequence (flip angle 40°, repetition time TR = 40 ms, echo time TE = 5 ms for each image). The brains were then embedded in paraffin and serially sectioned (20 μm) in the coronal plane (Fig. 2a−c). Sections were mounted on gelatin-coated slides and stained with a silver method for cell bodies (Merker 1983). Every 60th section (corresponding to the obtained blockface images, spacing 1.2 mm) were analyzed cytoarchitectonically, and used for the 3D reconstructions of the brains. The methods have been described in detail elsewhere (Amunts et al. 2000).

### Cytoarchitectonic analysis and definition of borders

Cortical borders were identified using multivariate statistical analyses of the laminar distribution of cell bodies (Schleicher et al. 1998, 1999, 2000, 2005). Therefore, rectangular regions of interest (ROIs) were defined in each histological section covering the dorsal occipital cortex (Fig. 2c). ROIs were scanned in a meander-like sequence using a CCD camera (XC-75, Sony, Tokyo, Japan, image matrix 736 × 544 pixel, in-plane resolution 1 μm per pixel), connected to a microscope (Planapo^®^ 6,3 × 1,25, Zeiss, Oberkochen, Germany), and a computer-controlled motorized stage.

The grey level index (GLI) was calculated in small measuring fields of 17 × 17 micrometers, and GLI-images were generated. The GLI is a measure of the volume fraction of cell bodies (Schleicher et al. 1999; Schleicher and Zilles 1990; Wree et al. 1982). The outer- (between layers I and II) and inner (between layer VI and the white matter) contour lines of the cortical ribbon were interactively drawn (Fig. 2d) using a MATLAB based script (MATLAB Version 7.0, MathWorks, Inc., Natick, MA, USA). Equidistant density profiles (GLI-profiles) were extracted along traverses perpendicular to the cortical layers. To compensate for variations in cortical thickness, the GLI profiles were standardized to a cortical depth of 100%.

The shape of each profile was quantified by a set of 10 features, i.e., a feature vector, comprising the following parameters: the mean GLI value, the cortical depth of the centre of gravity, the standard deviation, the skewness and the kurtosis of the profile as well as the analogous parameters from the absolute value of its first derivative (Zilles et al. 2002). To detect changes in laminar pattern along the cortical ribbon, blocks of 8−24 neighboring profiles were compared using a sliding window procedure. A multivariate distance measure, the Mahalanobis distance, was calculated between neighboring blocks of profiles as an estimate of the differences in the laminar pattern between these blocks, i.e., differences in cytoarchitecture (Fig. 2f). The statistical significance of cytoarchitectonic differences was tested with a Hotellings *T* ^2^-test and Bonferroni corrected for multiple comparisons (*P* < 0.05). The positions of maxima were accepted as borders if found at comparable positions in neighboring sections and confirmed by visual inspection of the cell-stained sections. Using this approach, two areas, hOc3d and hOc4d were mapped in serial histological sections in both hemispheres of ten brains.

### Volumetry

The volumes *V* of the identified areas were calculated based on areal measurements on images of the histological sections:

$$V = s \cdot T \cdot \Updelta x \cdot \Updelta y \cdot F \cdot \sum N_i. $$

The resolution of images was 1,200 dpi; the spacing between two measured sections (*s*) was 60. The section thickness (*T*) was 0.02 mm. The pixel size (∆*x* and ∆*y*) was 0.0212 mm × 0.0212 mm. *F* is the individual shrinkage factor of the brain, and *N*_*i*_ the number of pixels of the cortical area in section *i* (Amunts et al. 2005). Areas were measured using in-house software in 17−29 sections through each of the two cytoarchitectonic areas, separately for each hemisphere and brain.

The volumes of areas hOc3d and hOc4d were analyzed with respect to inter-hemispheric (side) and inter-areal differences using an analysis of variance (ANOVA) with the following design: between subject factor ‘gender’ (male, female), within factors ‘hemisphere’ (left, right), ‘area’ (hOc3d, hOc4d) and blocking factor ‘brain’. In addition, the volumes were compared between hemispheres using paired *t* test. In all tests, the level of significance was set to *P* < 0.05.

### 3D reconstruction of post-mortem brains, generation of continuous probabilistic cytoarchitectonic maps and a maximum probability map (MPM)

The histological sections of the ten brains were 3D reconstructed using the blockface images of the paraffin-embedded brains, and the MR scans of the fixed brains taken prior to histological processing (Amunts et al. 2000). All data-sets were registered to the stereotaxic space of the T1-weighted single subject template of the Montreal Neurological Institute (MNI) reference brain (Collins et al. 1994; Holmes et al. 1998) using a combination of linear affine transformation, grey level normalization and non-linear elastic registration algorithms (Amunts et al. 2000; Hömke 2006).

Since the spatial resolution of the MRIs (fixed brain and in vivo reference brain ≈ 1 mm) is significantly lower than that of the histological sections (≈20 μm), images of the sections containing the contour lines of the delineated areas, hOc3d and hOc4d were downscaled. We applied a box filter, which assigned the fractional amount of the corresponding area in the original image to each data point in the downscaled image. Downscaled images were stored and further processed in a real number image format. Linear and nonlinear elastic transformations were used to 3D-reconstruct the histological data, and to register them to the reference brain. The different steps were assembled to minimize interpolation artefacts. For the residual interpolation, we used a sixth order *b*-spline interpolation (Thévenaz et al. 2000). The mean of the two downscaled and transformed areas of all ten brains was calculated and superimposed to the reference brain thus resulting in continuous probability maps scaled from 0.0 (=0%) to 1.0 (=100%). The maps show the probability, at which each area was represented in each voxel of the reference space, i.e., the probability quantifies the degree of interindividual microstructural variability in each voxel.

The volume data sets were then transferred to the anatomical MNI space, which has its origin at the upper edge of the anterior commissure (Amunts et al. 2005). The anatomical MNI space differs from MNI space by 4 mm in *y*-direction and 5 mm in *z*-direction. The anatomical MNI space has, in contrast to the MNI space, the same origin as the Talairach space (Talairach and Tournoux 1988).

A “maximum probability map” (MPM) of all early dorsal visual areas was calculated by analyzing the probabilities of all previously delineated occipital area (i.e., the numbers of overlapping representations) in each voxel (Eickhoff et al. 2005, 2007). Each voxel was assigned hereby to the most likely area. If different areas showed equal probabilities in the same voxel, this voxel was assigned to the area with the highest average probability in the 26 directly adjacent voxels. Volumes and positions of the two identified areas in the MPM representations were subsequently compared with the corresponding mean values of volumes.

## Results

### Cytoarchitecture of areas hOc3d and hOc4d

**Fig. 3:**
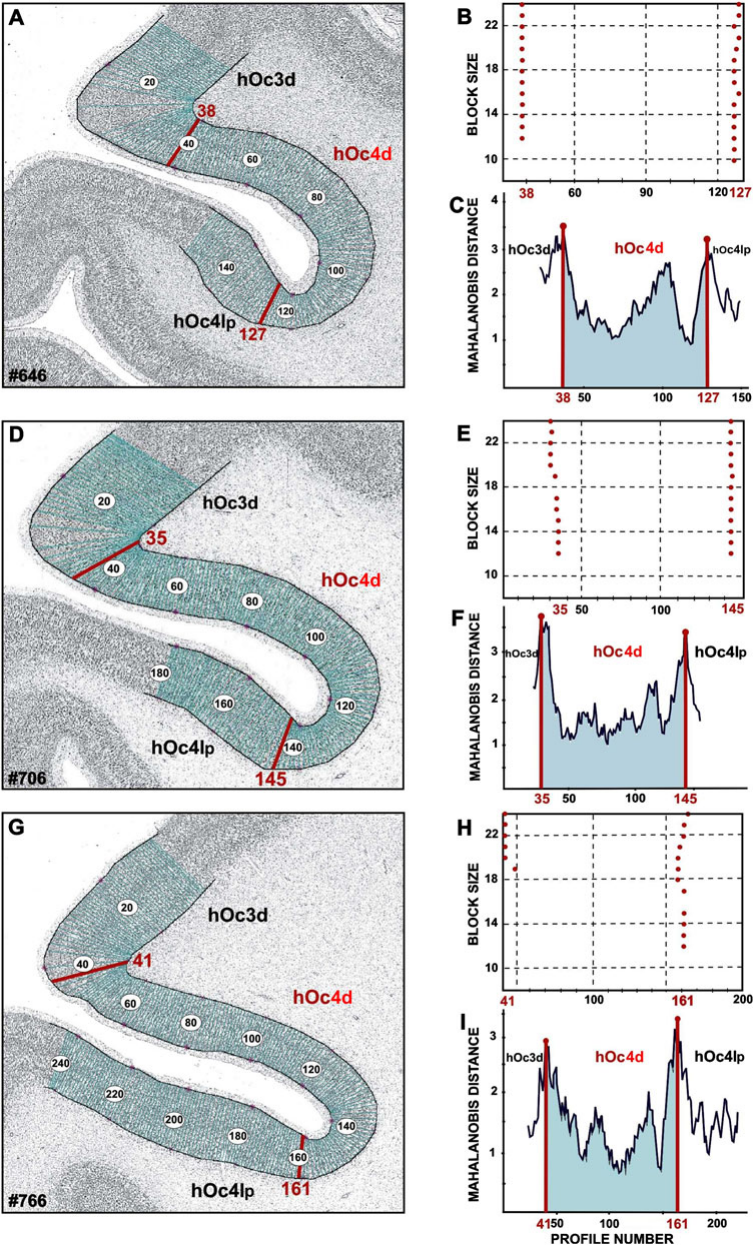
Positions of maxima in the region of the transverse occipital sulcus (TOS) in three adjacent, histological sections of brain number 7. Equidistant profiles were extracted from the layer I/II border to the layer VI/white matter border (**a**, **d**, **g**). The Mahalanobis-distance functions were calculated for the block size **b** = 8 until **b** = 24 (**b**, **e**, **h**). Significant maxima were found on the positions 38 (**a**, **b**), 35 (**d**, **e**) and 41 (**g**, **h**; *P* < 0.001). These positions, found in three adjacent histological sections, correlate with the borders between areas hOc3d and hOc4d, which could also be identified by visual inspection of this sections. The border between area hOc4d and adjacent to the posterior lateral occipital area hOc4lp was identified in the same way. Significant maximum was found at position 127 (**a**, **b**) in section number 646. This position correlates with the maxima found at the positions 145 (**d**, **e**) in section number 706, and 161 (**g**, **h**) in section number 766. Mahalanobis distance functions calculated for the block size 24 are plotted in (**c**, **f**, **i**)

Two new cytoarchitectonic areas, hOc3d and hOc4d, were delineated in the human dorsal extrastriate cortex; they were located lateral and dorsal to BA 18/V2. Cytoarchitectonic borders of the region were defined based on quantitative criteria (Schleicher et al. 1999) in neighboring sections in all ten brains (Fig. 3). In total, more than 200 ROIs were analyzed.

**Fig. 4:**
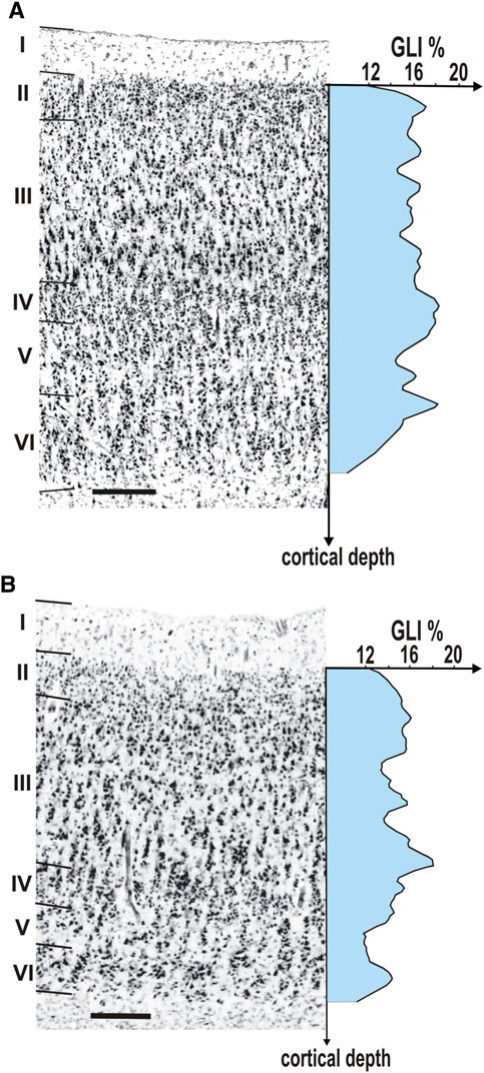
Cytoarchitecture of areas hOc3d (**a**) and hOc4d (**b**) with the respective GLI profiles (*x*-coordinate: cortical depth, *y*-coordinate: GLI value in %). **a** Area hOc3d is characterized by a broader layer V and an inconspicuous layer VI. **b** Area hOc4d shows conspicuous pyramidal cells in layer IIIc, thin layers V and VI and a better defined cortex/white matter border. *Roman numerals* indicate cortical layers. *Scale bar* 250 μm

The microstructural differences between areas hOc3d and hOc4d can be summarized as follows. Area hOc3d was characterized by a relatively uniform cell density, which is reflected by GLI profiles with a relatively local course (Fig. 4a). In addition, the pyramidal cells in layer III slightly increased in size towards layer IV. Area hOc4d was characterized by a conspicuous layer IIIc with large pyramidal cells, which corresponded to a distinct local maximum in the GLI profile (Fig. 4b). The thickness of the infragranular layers V and VI was smaller in hOc4d than in hOc3d, and the cortex/white matter border was sharper in hOc4d as compared to area hOc3d.

The cytoarchitecture of area hOc3d differed from that of the adjacent BA 18/V2 (Amunts et al. 2000) by a lower cell-packing density in layers II and IIIa, and smaller size of pyramidal cells in sublayers IIIb and IIIc. Layer IV was found at a more superficial position in area hOc3d than in BA 18/V2. The pyramidal cells in sublayer Va of hOc3d were larger than those of BA 18/V2. Furthermore, layers V and VI of hOc3d did not contain cellular clusters typical for BA 18/V2. The neurons of hOc3d showed a more pronounced radial cellular arrangement comparing to area BA 18/V2 [“rain showers” (von Economo and Koskinas 1925)].

**Fig. 5:**
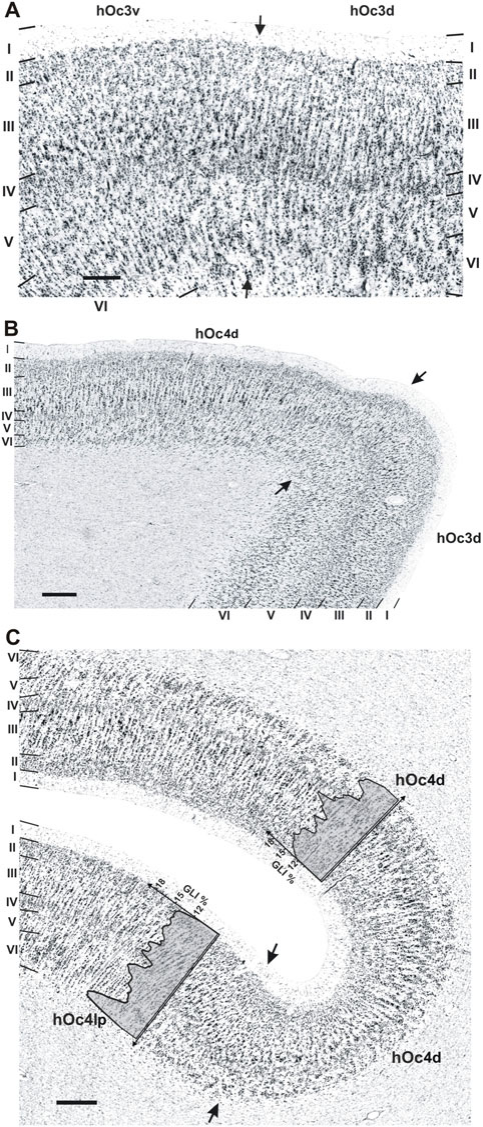
**a** Cytoarchitectonic border between area hOc3d and area hOc3v (indicated by *arrows*). The border is characterized by the larger pyramidal cells in sublayers IIIb and IIIc in area hOc3v than in area hOc3d, and by a less obvious columnar arrangement in hOc3v compared to hOc3d. *Roman numerals* indicate cortical layers. *Scale bar* 250 μm. **b** Border between areas hOc3d and hOc4d (indicated by *arrows*). These areas differed from each other mainly by the size of pyramidal cells in lower layer III (more conspicuous in area hOc4d). Furthermore, layers V and VI are thinner in area hOc4d, and the layer VI/white matter border is better defined in area hOc4d. *Scale bar* 500 μm. **c** Border between area hOc4d and area hOc4lp (indicated by *arrows*); typical GLI-profiles for each area are given (*x*-coordinate: cortical depth, *y*-coordinate: GLI value in %). This border is characterized by a decrease in the size of pyramidal cells in sublayers IIIb and IIIc, a narrower layer III in area hOc4lp, and a smaller layer III in area hOc4lp. Furthermore, layers V and VI are considerably thicker in area hOc4lp, and the cortex/white matter border is less well defined in area hOc4lp than in area hOc4d. *Scale bar* 500 μm

In the occipital pole region, area hOc3d bordered not only on BA 18/V2, but also on area hOc3v of the ventral visual cortex (Rottschy et al. 2007). The external granular cell layer (layer II) had a lower cell-packing density in hOc3d than in hOc3v (Fig. 5a). The gradual increase in the size of pyramidal cells from sublayer IIIa to IIIc was seen in both areas (hOc3d and hOc3v), but the number of large pyramidal cells in sublayer IIIc was higher in area hOc3v than in area hOc3d. Layer IV of area hOc3v was more cell-dense, and had a less sharp border to layer V. The contrast in cell density between layers V and VI was higher in hOc3v than in hOc3d but the size of pyramidal cells in sublayer Va was larger in area hOc3d. Area hOc3d bordered on area hOc4d along its caudal and medial parts (Fig. 5b).

The lateral part of area hOc4d bordered on a region which we labeled as area hOc4lp (posterior lateral occipital area). Areas hOc4d and hOc4lp differed from each other by the volume fraction of cell bodies in layers II and IIIa (lower in hOc4lp), the size of pyramidal cells in sublayers IIIb and IIIc (smaller in hOc4lp) and the thickness of layer III (lower in hOc4lp) (Fig. 5c). Furthermore, layers V and VI were considerably thicker in area hOc4lp, and layer V had a higher cell-packing density in this area than in hOc4d. Layer VI in area hOc4lp was more cell-sparse, and the cortex/white matter border was less clearly defined in hOc4lp than in hOc4d.

### Topography of the areas

**Fig. 6:**
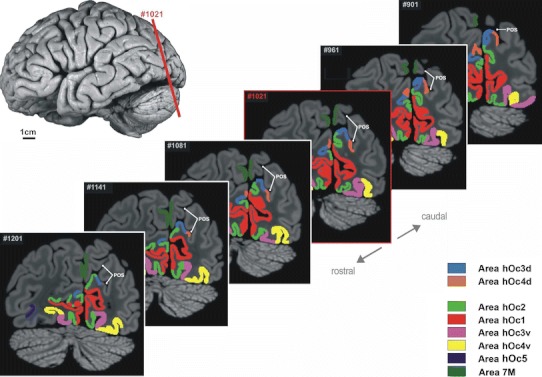
Mapping of areas hOc3d and hOc4d as well as their neighboring areas (Amunts et al. 2000; Malikovic et al. 2007; Rottschy et al. 2007; Scheperjans et al. 2008) in six MRI coronal sections of an individual post-mortem brain. The *red line* in the upper left image shows the position of section 1021. Identified areas are labeled by different colors. *POS* parieto-occipital sulcus

Area hOc3d bordered on BA 18/V2 in all histological coronal sections. The border between areas hOc3d and BA 18/V2 was found on the lateral surface in the most caudal sections. In the more rostral sections, this border was located at the anterior part of the cuneus in the posterior (occipital) bank of the POS. Area hOc3d was located immediately lateral to the dorsal part of BA 18/V2 (Fig. 6). Three anatomical landmarks were defined: the superior occipital sulcus (SOS), the transverse occipital sulcus (TOS), and the parieto-occipital sulcus (POS). Area hOc3d extended in part along the lateral surface of the occipital lobe in close relationship to the SOS. The anterior part of this sulcus crossed area hOc3d along the rostro-caudal direction.

Next to area hOc3d, area hOc4d was identified. It bordered on area hOc3d along its full length. Area hOc4d was located more rostral and lateral than area hOc3d in all of investigated hemispheres (Fig. 6). It was closely linked to the TOS, which presents a landmark for the rostral end of hOc4d. In most of cases, it was interposed between the SOS and the superior margin of the occipital lobe. Both areas (hOc3d and hOc4d) occupied the dorsal portion of the occipital lobe, and extend to its medial and lateral surface. In the most caudal sections, both areas were located on the lateral surface of the occipital lobe with a smaller portion on its medial surface. In the most rostral sections, both areas always occupied the medial surface of the occipital lobe where they extend along the posterior (occipital) bank of the parieto-occipital sulcus.

### Volumes of areas hOc3d and hOc4d

**Fig. 7:**
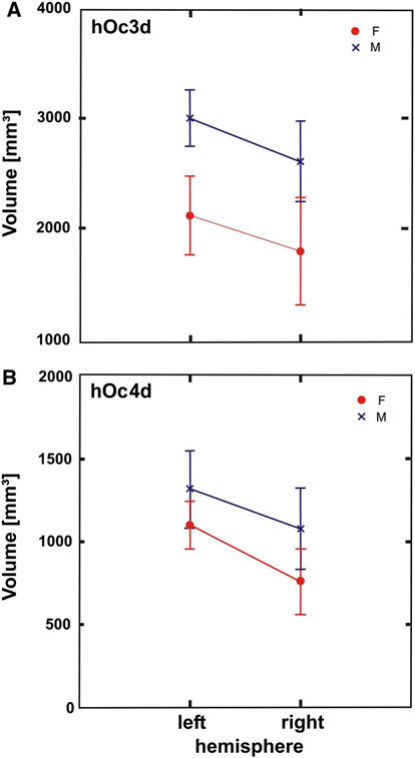
Statistical analysis (means ± SD) of the volumes (separated by hemispheres) for males (*blue*) and females (*red*) of areas hOc3d (**a**) and hOc4d (**b**)

The mean total volume (both sides taken together) was significantly larger in area hOc3d than in area hOc4d (ANOVA, *P* < 0.0001, Fig. 7). The ANOVA indicated a significant effect of side if both areas were taken together (*P* < 0.05), but no interactions were found between side and area. Male and female brains did not differ in volume. No interaction of gender with other factors was found. There was a tendency of a larger volume of the areas in male than in female brains (hOc3d and hOc4d), but due to the large variance the difference did not reach significance (*P* > 0.05, Fig. 7).

The left mean volume of area hOc3d was 2,567 ± 736 mm³ (mean ± SD) compared to 2,209 ± 897 mm³ in the right hemisphere; this difference was not significant (*P* > 0.05). Eight of ten brains showed a larger volume of area hOc3d in the left than in the right hemisphere. The mean volume of area hOc4d was significantly larger (*t* = 2.8975, df = 9, *P* < 0.05) on the left side (1,208 ± 372 mm^3^) than on the right side (975 ± 443 mm^3^). Seven of ten brains had a larger mean volume in the left than in the right hemisphere.

### Continuous probabilistic maps and stereotaxic localization

**Fig. 8:**
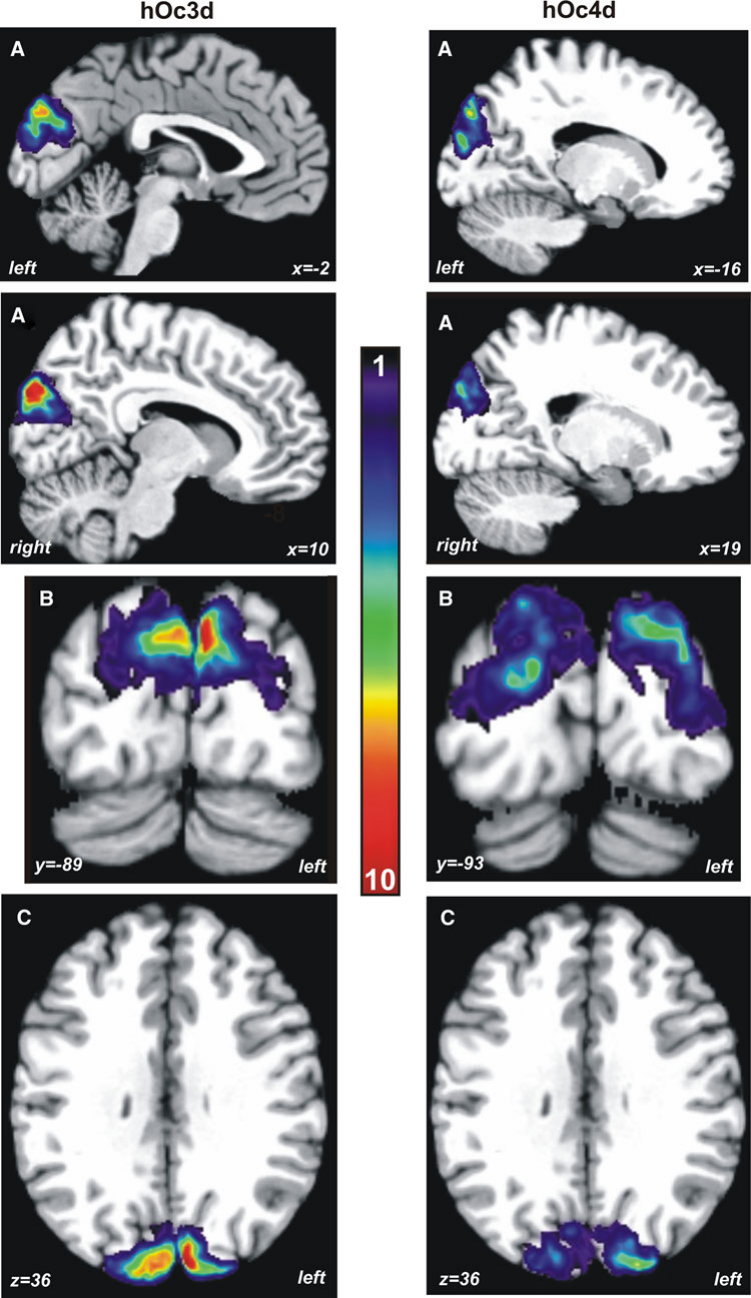
Continuous probabilistic maps of areas hOc3d and hOc4d in anatomical MNI space. The number of overlapping brains in each voxel of the reference brain is color-coded from *dark blue* (1 of the 10 brains was present in this voxel) to *dark red* (overlap of all 10 brains). Note the higher interindividual variability of area hOc4d as compared to hOc3d. **a** Sagittal, **b** coronal, **c** horizontal plane

Continuous probabilistic cytoarchitectonic maps of both areas were generated in anatomical MNI reference space (Fig. 8). They were color coded in a smooth spectral sequence from dark blue (lowest areal presentation) to dark red (highest areal presentation). The maps showed a high intersubject variability of both areas, which was particularly obvious in area hOc4d. In this area, no voxels were found with more than eight overlapping brains. Area hOc3d showed an overlap of 10 brains. This variability in overlap is also reflected in the high variability of the individual centers of gravity as shown in Table 2. While area hOc4d was always found laterally and rostrally to area hOc3d, the centers of gravity across all subjects lay within one standard deviation of each other.

**Table 2:** Coordinates (centres of gravities) of areas hOc3d and hOc4d in anatomical MNI space (mean ± SD across the 10 brains after nonlinear normalization).* L* – left;* R* – right;* X* – sagittal plane;* Y* – coronal plane;* Z* – horizontal plane. Data in millimeters

Area	Anatomical MNI space		
	*X*	*Y*	*Z*
hOC3d_L	−15 ± 5	−97 ± 4	23 ± 7
hOC3d_R	17 ± 5	−95 ± 2	24 ± 6
hOc4d_L	−17 ± 6	−95 ± 4	29 ± 7
hOc4d_R	19 ± 4	−94 ± 3	29 ± 6

**Fig. 9:**
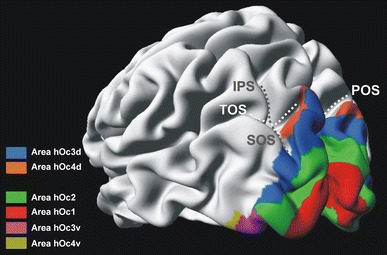
Maximum probability map of areas hOc3d and hOc4d as well as their neighboring areas (Amunts et al. 2000; Rottschy et al. 2007) on the inflated surface of the MNI single subject template. *IPS* intraparietal sulcus, *POS* parieto-occipital sulcus, *SOS* superior occipital sulcus, *TOS* transverse occipital sulcus

The maximum probability map (MPM) reflects the most frequent positions of each area in a sample of ten post-mortem brains. Figure 9 shows this pattern of areas hOc3d and hOc4d as well as their adjacent areas (Amunts et al. 2000; Rottschy et al. 2007) in the region of the occipital lobe. The data were displayed on the cortex/white matter surface of the MNI single subject template.

## Discussion

Functional imaging studies allow the delineation of primary and higher visual areas based on retinotopic mapping, a non-invasive method used to define the borders of visual areas during phase-encoded retinal stimulation (see below). For many of areas that have been delineated using these methods, the correlating cytoarchitectonic areas have not yet been identified. Observer-independently, borders of two new areas of the dorsal visual cortex were identified in the present study: hOc3d and hOc4d were mapped based on quantitative, reproducible criteria. This analysis of cytoarchitectonic differences allows a reliable delineation between areas hOc3d and hOc4d, as well as from adjacent areas in a region, that was not further parcellated by Brodmann (1909) and the investigators following him.

### Comparison with previous anatomical maps

Brodmann’s map (1909) shows a relatively simple subdivision of the human visual cortex into the three areas BA17, BA18, and BA19. Our areas hOc3d and hOc4d occupy parts of the presumed BA 19, and may even exceed it. Since Brodmann did not report the positions of area 19 in relation to sulci in sufficient detail, and did not investigate the putative subdivision of this region, a comparison with his maps is hardly feasible. Moreover, the concept of a simple tripartion of the visual cortex as shown in his and other studies is obsolete considering recent functional imaging studies in the human brain as well as functional and anatomical studies in the macaque brain (see below).

The cytoarchitectonic analysis and description of Area peristriata (OA; von Economo and Koskinas 1925), which roughly corresponds to BA 19, was more detailed and allowed a more specific cytoarchitectonic comparison with the data of the present study. Von Economo and Koskinas found that Area peristriata (OA) was structurally heterogeneous; they identified three subdivisions (OA_1_, OA_2_, OA_M_). The first two areas are located dorsally, the third more ventrally (Fig. 1c). The description of von Economo and Koskinas points towards a dorso-ventral subdivision of OA although they did not define the subdivisions as areas, but rather local modifications of OA. OA_1_ (Fig. 1c, d) may correspond with respect to its localization to our area hOc3d, although cytoarchitectonically we identified a prominent sublayer IIIc and a wider lamina V, which was not described in von Economo and Koskinas (1925) paper. OA_2_ (Fig. 1c, d), in which the size of the pyramidal cells increases comparing to adjacent areas, may correspond to our area hOc4d by localization. The description of OA_2_ by von Economo and Koskinas (1925) does not enable an unambiguous identification of the areas, and quantitative data of the laminar distribution of cells were not available.

Areas hOc3d and hOc4d are located in the region of Area peristriata cunealis medialis and Area peristriata cunealis lateralis as defined by Braak (1977), who analyzed the pigment- and cytoarchitecture. Although the cytoarchitecture of Braak’s Area peristriata cunealis medialis can be well compared to our area hOc3d, the topographic comparison is more difficult. Area hOc3d overlaps with parts of Area peristriata cunealis medialis and Area peristriata cunealis lateralis. It can be assumed that the area hOc4d is approximately located in the rostral half of the Area peristriata cunealis medialis.

The present study revealed significant cytoarchitectonic differences between areas hOc3d and hOc3v, an area of the ventral visual cortex (Rottschy et al. 2007). Thus, there are two areas adjacent to BA 18/V2, which is in accordance to earlier observations. Eickhoff et al. (2008) revealed a dorso-ventral differentiation based on the analysis of the distribution of different receptor types of classical neurotransmitter system. They have found significant differences between area hOc3v and hOc3d with higher receptor density for five receptor types (5-HT_1A_, α_2_, GABA_A_, M_1_ and NMDA) in hOc3v as compared to hOc3d. Since transmitter receptors are key molecules of neurotransmission (Zilles and Amunts 2009), this data supports the hypothesis of a functional difference between the dorsal and the ventral part of the human visual cortex rostral to BA 18/V2. Furthermore, dorso-ventral differences in connectivity have been demonstrated by a tracing study of callosal connections (Clarke and Miklossy 1990). A “dorsal area V3” was located in the upper part of the occipital lobe, more lateral and dorsal than “area V2”. The position of “dorsal V3” roughly corresponds to the position of our area hOc3d based on the published images of sections, although Clarke and Miklossy did not provide a three-dimensional map of this area with anatomical landmarks. The same is true for the border to the adjacent area V3a. Due to the cytoarchitectonic and myeloarchitectonic differences authors proposed the separation of area V3 onto dorsal (“dorsal V3”) and ventral (“VP”) part, similar to the separation in macaque monkeys (Burkhalter et al. 1986). These findings are in good agreement with our results.

### Comparison with functional imaging data

Retinotopic mapping of cortical responses to localized stimuli is an important tool for the delineation of visual areas in the brain of non-human primates using electrophysiologic recordings (Essen and Zeki 1978) and functional neuroimaging (Brewer et al. 2002). Given the clear topological layout of visual responses, the presence of a distinct retinotopic map has in fact been suggested as a key criterion for the definition of a distinct area (Orban et al. 2004). A similar pattern of retinotopic organization within the human visual system was revealed using PET (Fox et al. 1987) and fMRI (Sereno et al. 1995), and has since represented the predominant approach to identify the location of human visual areas in vivo*.* The total number of retinotopic representations is still a matter of debate (Arcaro et al. 2009, 2011). Nevertheless, comparative studies revealed a close correspondence of retinotopic maps for the early visual cortex with data from non-human primates (Van Essen et al. 2001; Orban et al. 2004), anatomical definitions (Wilms et al. 2010), functional connectivity (Heinzle et al. 2011), or TMS effects (McKeefry et al. 2009). Based on their location adjacent to the dorsal portion of V2 and their spatial relation to the SOS, it may hence be assumed that hOC3d and hOC4d correspond to functionally defined areas V3d and V3A, respectively. Area V3d represents the contralateral lower quadrant of the visual field and if combined with V3v (hOC3v) a complete representation of the visual field. In spite of this joint visual field representation, distinctions between both can be found on the molecular and functional level (Eickhoff et al. 2008), which may reflect behavioral advantages for the lower visual field in tasks such as visuomotor feedback processing (Khan and Lawrence 2005), visually guided pointing (Danckert and Goodale 2001) and spatial relocation memory (Genzano et al. 2001). The amount to which such differences are reflected in functional response properties as assessed using fMRI, however, has been disputed. Whereas some authors do advocate clear differences (Kraft et al. 2011), other authors have failed to replicate these, and consider the two fields as parts of the same area (Wade et al. 2002; Zeki 2003). Given the conspicuous anatomical differences between hOC4v and hOC3d, however, both areas should probably be considered anatomically distinct yet functionally closely related areas. Functionally, Area V3A, which contains a full representation of the contralateral hemi-field (comparing with neighboring area V3d that contains only a map of the contralateral quadrant), has been shown to be involved in the perception of stereoscopic (Anzai et al. 2011; Tootel et al. 2003) and chromatic motion (McKeefry et al. 2010). The latter study pointed out that there is a minor effect on perceived speed of motion stimuli when areas V3d and V3B are activated compare to areas V3A and V5/MT+. V3A (and LOC) moreover seem to encode figure-ground relationships and object convexity (Cottereau et al. 2011). In line with this view, it has been shown, that V3 and V3A may be involved in the extraction and processing of the 3D shape (Georgieva et al. 2009). V3A is also differentially activated by the degree of contour curvature in stationary and moving forms (Caplovitz and Tse 2007). Other data, however, seemed to contradict such interpretation and the respective authors therefore stressed the role of V5/MT+ in that respect (Harvey et al. 2010). It has thus been proposed, that V3A may be involved less in the perception but rather the prediction of visual motion (Maus et al. 2010). Given this rather diverse functional interpretations, it may be justified to acknowledge, that the precise computational mechanisms and hence the processes supported by V3A are yet unknown. The concept of a “V3A complex” has been raised (Georgieva et al. 2009), which is supposedly located near the transverse occipital sulcus and consists of at least two rostral (V3C and V3D) and two caudal subdivisions (V3A and V3B). While the current anatomical definition of hOc4d seems to correspond roughly to the entire “V3A complex”, the precise correspondence between the variably defined (in terms of their nomenclature and functional characteristics) functional entities in that region remains to be further investigated.

The comparison of the retinotopic map for area V3d with cytoarchitectonic data of hOc3d showed a close correspondence between anatomical and functional definitions, which is in line with the close convergence found in the remaining early human visual areas (Wilms et al. 2010). Whereas, our area hOc3d corresponds to functionally defined area V3d, our area hOc4d shows maximal overlap with V3A. Cytoarchitectonically, hOc3d and hOc4d are indeed very similar to each other, even more similar than the ventrally located areas hOc3v and hOc4v (Amunts et al. 2007). In contrast to a previous cytoarchitectonic study (Amunts et al. 2000), we here decided to apply a neutral nomenclature, and named the areas according to their appearance as areas #3 and #4 when moving from (primary) area 17/V1 in dorsal direction (hOc3d and hOc4d). We do not wish, at this point, to imply a hierarchy among the occipital visual areas although the match with the nomenclature of some functional imaging data is less obvious. Moreover, different concepts underlying areas, subareas and subdivisions, regional specializations, etc. have been inconsistently used in past, and the concept of a “subarea” is vaguely defined. Therefore, we adopt the terms hOc3d and hOc4d for the two areas of the dorsal visual cortex throughout the study.

### Spatial localization and interindividual variability

The localization and extent of both areas (hOc3d and hOc4d) can be characterized in relation to the three occipital sulci POS, SOS, and TOS. However, intersubject variability and interhemispheric differences of sulci have to be taken into account (Elliot Smith 1904; von Kuhlenbeck 1928; Malikovic et al. 2011; Ono et al. 1990; Filimonoff 1932). The the parieto-occipital sulcus (POS) is a continuous sulcus which runs along the medial surface of the hemisphere from the superior margin of hemisphere to the cuneal point (the spot where the POS meets the calcarine sulcus). The POS is very deep and its shape can vary considerably (straight, *X*-form, *Y*-form or arborescent form). In 96% of the cases, the dorsal (upper) part of the POS extends further along the lateral surface of the hemisphere in the region of parieto-occipital transition (Ono et al. 1990). In our sample, the POS was reached the lateral surface in all 20 hemispheres. Area hOc3d was located on the posterior (occipital) bank of the POS in all cases. This finding is in agreement with the position of area V3 of the macaque brain; it lies together with area V2 on the posterior bank of the POS (Gattass et al. 1981, 1988). Area hOc4d was always identified along this bank of the POS, but more dorsal than area hOc3d.

The superior occipital sulcus (SOS) is frequently found as an extension or side branch of the intraparietal sulcus (IPS). The SOS extends rostro-caudally along the dorsal part of the occipital lobe and often intersects the transverse occipital sulcus (TOS) at a right angle. The position of area hOc4d is comparable to that of area V3A as shown by Tootell et al. (1997) and McKeefry et al. (2010). Their studies demonstrated that area V3A was located in the TOS and the superior occipito-parietal region. This is in good agreement with our observations where area hOc4d was found in the TOS, which was seen in 90% of the cases. In all cases, area hOc4d was located in the region of the SOS-TOS intersection. In the most caudal sections, hOc3d and hOc4d extend on the lateral hemispheric surface, whereby area hOc4d was always more rostro-laterally positioned. At rostral levels, both areas moved to the medial surface of the occipital lobe. This extension was present in all hemispheres, and is in agreement with the topology shown in various retinotopic studies (DeYoe et al. 1996; Logothetis 2002; van Essen and Drury 1997). In comparison to area hOc4d, area hOc3d had the longer axis in the dorso-ventral direction and was found at more caudal sections through the occipital lobe. This topology is comparable with functional data of areas V3d (“dorsal V3”) and V3a presented by Harvey et al. (2010).

The warping of the individual areas to a common reference space eliminates the interindividual variability of the external brain morphology, but the interindividual variability in localization and extent of cytoarchitectonic areas remained. For example, area hOc4d is located along the anterior bank of the TOS in most of cases, it may be located along the posterior bank of the TOS in approximately 10% of cases. Another example of interindividual variability is the location of area hOc3d along the lateral surface of the occipital lobe. In most of cases, area hOc3d has an inferolateral position when it extends below the SOS along the caudal parts of the lateral occipital sulcus. Rarely, area hOc3d has a superolateral location when it extends between the SOS and the superior margin of the occipital lobe. The latter topography was found in 20% of cases (4 hemispheres).

The probabilistic maps showed a relatively high intersubject variability in the spatial extent of both areas with hOc4d being more variable than hOc3d (Fig. 8). This variability is influenced by the large variability of the volumes of the areas (e.g., smaller areas more variable than larger areas), but also by methodological factors (e.g., elastic spatial normalization). The volume of area hOc4d was smaller than that of hOc3d which is in line with this line of argumentation. In addition, increasing deviation of the areal shape from that of a sphere increases the influence of methodological factors (registration of the borders, smoothing and interpolation of the 3D-data) and their effects regarding to the overlap in the probability map (Malikovic et al. 2007).

The localization of those parts of the probability maps of the individual brains, which showed a high overlap (low variability, corresponding approximately to the maximum probability maps), coincides nicely with the localization of the area found in every individual brain. The cytoarchitectonic probabilistic maps can be directly compared with functional activations obtained by in vivo imaging studies, improves the anatomical interpretation of such data, and elucidates the interrelationship between structure and function. The maps are available for download from the Juelich website (http://www.fz-juelich.de/inm/inm-1/EN/Service/Download/download_node.html and http://www.fz-juelich.de/inm/inm-1/DE/Forschung/_docs/SPMAnantomyToolbox/SPMAnantomyToolbox_node.html), and as part of different software tools and databases, e.g., the SPM toolbox (Eickhoff et al. 2005), FSL and AFNI.
